# Multiplexable, High-Throughput DNA-Based Technologies in Screening and Confirmatory Testing of Newborn Conditions: A Scoping Review

**DOI:** 10.3390/ijns11040104

**Published:** 2025-11-13

**Authors:** Terence Diane Fabella, Joery den Hoed, Lidewij Henneman, Wendy Rodenburg, Johannes C. F. Ket, Jan Schouten, Erik A. Sistermans

**Affiliations:** 1 Department of Human Genetics, Amsterdam UMC, Location Vrije Universiteit Amsterdam, 1007 MB Amsterdam, The Netherlands; t.d.fabella@amsterdamumc.nl (T.D.F.);; 2 Amsterdam Reproduction and Development Research Institute, 1100 DD Amsterdam, The Netherlands; 3 MRC Holland, 1057 DL Amsterdam, The Netherlands; 4 Centre for Health Protection, National Institute for Public Health and the Environment (RIVM), 3721 MA Bilthoven, The Netherlands; 5 Medical Library, Vrije Universiteit Amsterdam, 1081 HV Amsterdam, The Netherlands

**Keywords:** newborn screening, next-generation sequencing, whole-genome sequencing, whole-exome sequencing, targeted sequencing, qPCR, MassARRAY, DNA-based technologies

## Abstract

Newborn screening (NBS) is evolving as novel technologies offer the opportunities to include a broader range of treatable disorders in its programs. Multiplexable, high-throughput DNA-based technologies such as next-generation sequencing (NGS) are being explored to improve and expand disease detection, although several issues have been raised with its use. This scoping review aimed to identify multiplexable, high-throughput, DNA-based technologies that were used for screening or confirmatory testing of newborn disorders in published studies. Available evidence on the appropriateness of technologies in the NBS context was extracted. A literature search (Medline, Embase, and Web of Science) was performed from inception up to April 2024 in collaboration with a medical information specialist. After selection, 26 journal articles were included that used these technologies for either screening (*n* = 12) or confirmatory testing (*n* = 14). Five technologies were identified: whole-genome sequencing, whole-exome sequencing, targeted gene sequencing (TGS), quantitative polymerase chain reaction, and MassARRAY. The majority used TGS (*n* = 19, 73.08%). The data extracted concern mainly technical aspects, and these suggest that a combined approach, i.e., testing via NGS plus a biochemical test, in parallel or reflex, emerges as the optimal option. Ethical and economic evidence is limited and rarely reported in the reviewed articles.

## 1. Introduction

The technological advancements in newborn screening (NBS), from testing for one disorder per test such as phenylketonuria in the 1960s to the introduction of tandem mass spectrometry in the 1990s, have resulted in screening of multiple inborn errors of metabolism in one run [1,2]. However, analytes measured by the traditional biochemical methods can be affected by several factors, such as nutrition, illness, gestational age, age at sample collection, and weight of the newborn at birth [3,4]. Additionally, for some conditions no metabolite is available for detection using the traditional NBS methods.

For conditions without metabolites to be used for screening by traditional methods, DNA-based methods such as qPCR and MassARRAY have been explored. qPCR has been used in detecting newborn conditions such as severe combined immunodeficiency disorder (SCID), spinal muscular atrophy (SMA), sickle cell disease, and X-linked agammaglobulinemia [5,6,7,8]. MassARRAY is a genotyping technique for detecting hundreds of genetic aberrations based on matrix-assisted laser desorption/ionization–time-of-flight mass spectrometry [9]. The technique involves site-specific PCR amplification and single-nucleotide extension into the variant site using a specific oligonucleotide primer and a terminator dideoxyoligonucleotide [10]. MassARRAY has been evaluated for screening of SMA [9], detection of *CFTR* causing variants in previously genotyped cystic fibrosis (CF) patients [11] and in patients with non-syndromic hearing loss [12] and Fabry disease [13].

The use of multiple parallel high-throughput DNA-based methods, such as next generation sequencing (NGS), is now being explored as well in NBS, as they enable simultaneous processing of a large number of samples and targeting of more genes to improve test performance and include more diseases [14]. NGS allows rapid, massively parallel sequencing of hundreds to thousands of genes [15]. Technically, it has three main sequencing approaches, whole-genome sequencing (WGS), whole-exome sequencing (WES) and targeted gene sequencing (TGS), wherein either the whole genome or only the coding regions of all genes and selected genes are sequenced, respectively [1]. Independently of the technology used, during analysis filters can be used to limit the scope to genes of interest. With NGS’s decreasing cost, the probability of its use in NBS programs is becoming more feasible [16]. About 30 international scientific research and commercial programs are sharing expertise and discussing effective strategies regarding the use of NGS in NBS through the International Consortium on Newborn Sequencing (ICoNS) [14].

With the increase in popularity of NGS in newborn pilot studies, challenges for implementation of these technologies in an NBS setting have been raised, including technical, ethical, and economic concerns [1,17]. Technical concerns include the capacity to handle high sample volume, time to result or turnaround time (TAT), analytical accuracy of tests, particularly on dried blood spot (DBS)-obtained DNA, interpretation of results in asymptomatic newborns, availability of independent methods for confirmation, and data storage [1,17]. Ethical issues reported are concerns about informed consent, inclusion of adult-onset conditions, potential misuse of genetic data, and the occurrence of inconclusive results such as variants of uncertain significance (VUS) and low-penetrance variants [1,17,18]. Finally, economic considerations include costs of the tests, including additional costs for computing, storage, and human resources, and cost-effectiveness.

This scoping review aimed to identify all multiplexable, high-throughput, DNA-based technologies that were used for screening and confirmatory testing of newborn disorders in published studies. Confirmatory testing was included, as it differs in neonatal screening setting and from the clinical setting. The suspected newborn for confirmatory testing, although flagged positive for a condition, is often asymptomatic. Furthermore, in a screening setting the chance of obtaining false-positive results is much higher compared to a clinical setting, and therefore test specificity is considered crucial. Finally, short turnaround times are also a foremost factor in confirmatory testing of newborns compared to a clinical setting.

In this review, the available evidence and gaps that support or negate the appropriateness of the identified technologies in screening and confirmatory testing were extracted. Our aim was to provide information that contributes to understanding the advantages and disadvantages of available DNA-based technology in the context of NBS and confirmatory testing. Gaps identified provide insights when exploring genetic technologies to be used in NBS and offer suggestions about aspects for evaluation that need further studies.

## 2. Methodology

### 2.1. Protocol

The reporting of this scoping review was in accordance with the reporting guidelines provided by the Preferred Reporting Items for Systematic Reviews and Meta-Analyses extension for Scoping Review (PRISMA ScR) [19].

The research question and requirement for the inclusion and exclusion criteria were based on the population, concept, context (PCC) framework for developing research questions for the scoping review [20].

*Population* pertains to apparently healthy newborns who underwent screening, e.g., first tier or second tier, and screened positive newborns who underwent confirmatory testing.

*Concept* is multiplexable, high-throughput DNA-based technologies.

*Context* is newborn screening and confirmatory testing within the newborn screening pathway or process in a public health setting.

### 2.2. Literature Search, Abstract and Title Screening

A comprehensive search was performed on the Ovid Medline, Embase, and Clarivate Analytics Web of Science Core Collection databases with the medical information specialist J.K. Abstract and title screening of articles was conducted independently using an AI-powered tool, Rayyan, by T.D.F. and J.H. and was based on the selection criteria set for articles. Discrepancies were resolved through discussion between T.D.F. and J.H. and consultation with L.H., E.S., and J.S.

### 2.3. Study Categorization

Categorization of journal articles was based on the intended use of identified multiplexable, high-throughput, DNA-based technology in the article, either for screening or confirmatory testing. Articles included under the screening category were those that screened predominantly apparently “healthy” newborns and studies intended for tier testing, e.g., first- or second-tier screen. Articles under the confirmatory category were those that diagnostically tested newborns screened or suspected positive for a specific condition prior to testing and where it was indicated that the newborns were recalled, referred for confirmatory testing or diagnosis, and/or a new set of samples was recollected. Under each category, articles were grouped based on the identified technology, i.e., WGS, WES, not based on the method of analysis, i.e., in silico gene filtering. Study categorization was recorded on an Excel file worksheet (Supplementary File S1).

### 2.4. Eligibility Criteria

Included articles were original manuscripts that: (1) used multiplexable, high-throughput, DNA-based technologies for screening of multiple conditions in healthy newborns, (2) confirmatory testing of multiple conditions in screened positive (suspected) newborns, and (3) were published until April 2024. These articles were (4) written in English and (5) with available full text upon thorough search. Excluded articles were those that (1) used multiplexable, high-throughput, DNA-based technologies for other purposes aside from newborn screening and confirmatory testing of screened positive newborns, such as for clinical diagnoses of children with suspected genetic disease (e.g., newborns in neonatal intensive care unit), disease predisposition (e.g., cancer predisposition study), carrier studies, preconception, preimplantation, perinatal/noninvasive prenatal testing, cascade testing, family, single/trio/singleton studies, case reports, case series, molecular autopsy testing (e.g., molecular autopsy to identify cause of death of newborns) and (2) for technology validation purposes. Articles regarding (3) screening and confirmatory testing that tested only one newborn condition were also excluded, as well as (4) conference/meeting proceedings, reviews, project overviews, surveys/interviews/letters, genotype–phenotype associations and cost–benefit studies.

### 2.5. Data Extraction

The authors, year, setting, technology used, brief description, number of samples, and classification of samples tested were extracted from the articles. For the assessment of the technical applicability of the identified technologies, information about sample sources used, turnaround time, specificity and sensitivity, types of genetic variants detected, reported technical limitations, and DNA and non-DNA-based tests used as complementary tests were collected. For the assessment of the ethical applicability of the identified studies, data on consent and categories of disorders screened (treatable, actionable, and late-onset disorders, among others) and types of variants detected (low-penetrance variants, VUS, carrier status) were tabulated. Finally, for the assessment of the economic applicability of technologies identified, information about cost and cost per test were extracted as well. Full-text screening of selected articles and data extraction were carried out by T.D.F. with the assistance of J.H.

## 3. Results

### 3.1. Literature Search and Selection Process

**Figure 1:**
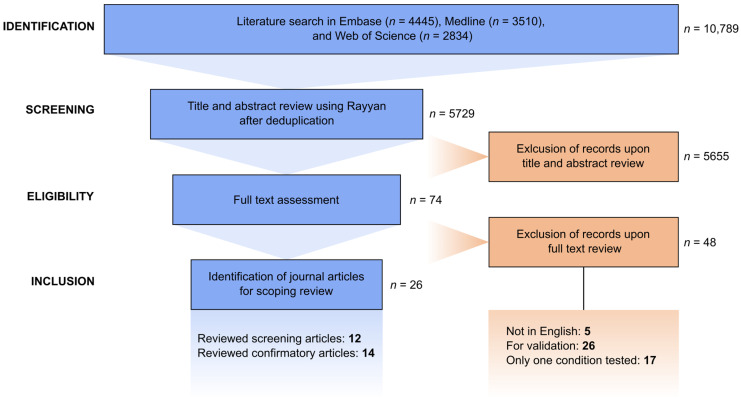
Flow diagram for the literature search and selection process based on the PRISMA-ScR guidelines for scoping reviews.

A comprehensive search was performed on the databases Ovid Medline, Embase, and Clarivate Analytics Web of Science Core Collection from inception to 18 and 19 April 2024 in collaboration with a medical information specialist, J.K. The search included controlled and free-text terms for synonyms of “newborn” or “infant” and “screening” and “high-throughput assays” or “whole-genome sequencing,” excluding “RNA” and “animal studies.” The search was performed without restrictions on methodology, date, or language. Full search strategies can be found in Supplementary File S2. Duplicate articles were excluded by J.K. using Endnote X20.0.1 (Clarivate^TM^). From the 5729 journal articles, 74 were selected as qualifying for full-text review, of which 26 were identified eligible for the scoping review. Twelve and fourteen articles were assigned under the screening and confirmatory categories, respectively. Figure 1 shows the entire screening and selection process.

### 3.2. Study Characteristics

**Figure 2:**
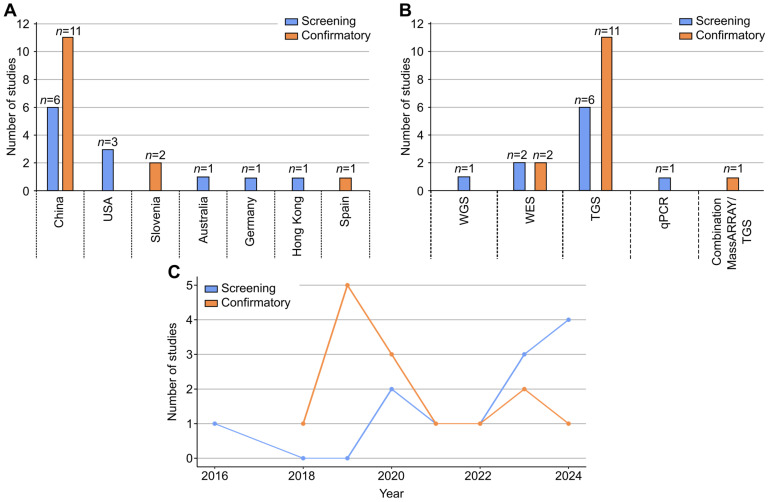
(**A**) Number of screening and confirmatory articles included per country. (**B**) Number of articles per technology under screening and confirmatory categories. (**C**) Number of published articles that used NGS technology under screening and confirmatory categories from 2016 to 2024.

The characteristics of the articles included are shown in Supplementary File S3 for the screening and confirmatory categories, respectively. Articles included were all original manuscripts of studies conducted from 2011 to 2023. The majority of the studies were conducted in China (*n* = 17, 65.4%), followed by the USA (*n* = 3, 11.5%), Slovenia (*n* = 2, 7.7%), Australia (*n* = 1, 3.9%), Germany (*n* = 1, 3.9%), Hong Kong (*n* = 1, 3.9%), and Spain (*n* = 1, 3.9%). The number of studies per country and category is shown in Figure 2A. The number of included participants in articles ranged from 106 to 96,015 for screening and 33 to 4809 for confirmatory testing.

Five different technologies were identified: TGS (*n* = 19), WES (*n* = 4), WGS (*n* = 1), multiplex qPCR (*n* = 1), and MassARRAY (*n* = 1, in combination with TGS). All NGS technologies detected used a short-read sequencing platform. For both screening and confirmatory categories, TGS, was the most common technology used (*n* = 19, 73.1%, Figure 2B).

There was an observed increase in the number of published screening articles that used NGS technology from 2022 to 2024. This increase was not observed for confirmatory articles (Figure 2C).

### 3.3. Dried Blood Spot (DBS) as a Source of gDNA

**Figure 3:**
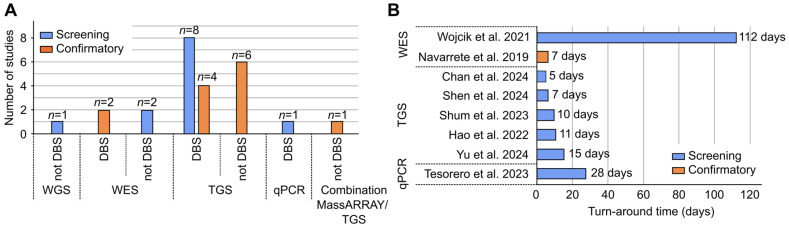
(**A**) Number of articles per technology that used and did not use dried blood spot (DBS) as a source of gDNA for testing. (**B**) Comparison of turnaround time (TAT), in days, of different technologies [8,23,31,32,33,34,35,36].

A total of 25 articles provided information about the source of genomic DNA (gDNA), while one article did not provide information. For the majority of articles (*n* = 15), DBS was used as a source of gDNA. The gDNA extracted from DBS was used for WES, TGS, and qPCR technologies (Figure 3A). The ten articles that did not report using DBS were those that used WGS (*n* = 1) [21], WES (*n* = 2) [22,23], TGS (*n* = 6) [24,25,26,27,28,29], and MassARRAY (*n* = 1) [30] technologies. These 10 articles used whole/peripheral blood, saliva, or oral epithelial cells as the source of gDNA for testing. The majority of the articles (*n* = 7, 70%) that did not use DBS as a source of gDNA for testing were technologies used in articles on confirmatory testing. The remaining three used non-DBS sources for screening (one WGS, two WES). For 6 (2 WES, 4 TGS) of the 13 confirmatory articles with available data on the source of gDNA, DBS was used as alternative source for peripheral whole blood for confirmatory testing.

### 3.4. Turnaround Time (TAT)

Eight articles (seven screening, one confirmatory) provided information about TAT. Figure 3B shows a comparison of the number of days needed from sample preparation to result generation using WES (7 and 112 days) [23,31], TGS (average of 10 days, ranged from 5 to 15 days) [32,33,34,35,36], and qPCR (28 days) [8]. No TAT data were provided for WGS or MassARRAY. For WES, TAT in screening was 105 days longer compared with confirmatory (Figure 3B).

### 3.5. Sensitivity, Specificity, Positive Predictive Value, and Negative Predictive Value

Only two screening articles provided data about the technology’s sensitivity, specificity, positive predictive value (PPV), and negative predictive value (NPV), and both were for TGS. Yu and colleagues compared four screening modes: independent biochemical (IBS), independent newborn sequencing (NeoSeq), sequential (SS), where samples were first tested with IBS and only newborns screened positive underwent NeoSeq, and combined screening (CS) [36]. They reported a sensitivity of 94.17%, specificity of 100%, PPV of 100%, NPV of 99.22% for independent NeoSeq and a sensitivity of 99.17%, specificity of 100%, PPV of 100%, and NPV of 99.89% for CS. Both NeoSeq and CS showed improved diagnostic metrics compared with IBS and SS, with reported sensitivity of 41.67% and 46.67%, specificity of 76.92% and 77.41%, PPV of 18.38% and 20.59%, and NPV of 91.13% and 92.04%, respectively. Shum and colleagues reported a sensitivity of >99% and specificity of 100% [34].

### 3.6. Technical Limitations of Identified Technologies in Terms of Variant Detection and Interpretation

All technologies detected single-nucleotide variants (SNVs) and small indels (Table 1), though for qPCR the SNV and deletion detected were limited to detection of the HbS pathogenic variant and *SMN1* exon 7 deletion only, respectively [8]. For WES, one article reported detection of large genomic rearrangements, although this technique does not allow determination of exact breakpoints or extent of the deletion [31]. For TGS, one article detected large copy number variants (CNVs), but involved using a special bioinformatic analysis package for CNV calling [34].

For all NGS technologies, WGS, WES, and TGS, multiple studies reported data quality limitations such as poor gene/s or exon/s coverage and the requirement for variant confirmation via Sanger sequencing (Table 1). For WGS and TGS, data analysis and interpretation variants detected in highly homologous genes and in genes with pseudogene were among the limitations reported. For WES and TGS, several studies reported that limited coverage to exonic regions only and restricted detection of large CNVs were among the commonly mentioned limitations (Table 1).

For qPCR and MassARRAY, no limitation was reported for data quality, analysis, or interpretation (Table 1). For both, limited genes and variants detected are considered a shortcoming. Hence, the authors of the articles recommended using these methods in combination with tandem mass spectrometry, TGS, and Sanger sequencing to improve sensitivity in disease detection.

To test specific genetic features that cannot be reliably detected by identified technologies, Sanger sequencing was the complementary method used most frequently to address technological limitations such as variant confirmation, determination of variant phasing, limited genes included in the panel, coverage limited to exonic regions only, and poorly covered exons due to subpar performance of NGS library generation. This was done by sequencing the newborn and its parents for the DNA regions that needed further evaluation (Table 1) [21,23,24,25,27,28,29,30,31,33,35,36,37,40,41,42,44,45]. On the other hand, MLPA was the method used to address the technological limitations regarding CNV detection [33,41,44] together with long-range PCR [32]. Clinical exome [21], droplet PCR [23], and Sanger sequencing [37] methods were also used for variant confirmation, while TGS was also used to address concerns regarding limited genes tested [30]. Other DNA-based methods used as complementary tests to technologies identified were custom probe design and the fluorescent PCR melting curve method for the detection of variants in highly homologous regions (Table 1) [35].

### 3.7. Complementary Non-DNA-Based Tests Used in Screening and Confirmatory Articles

Five screening articles used MS/MS and fluoroimmunoassays [8,33,36,39,40] in parallel with WGS, WES, TGS, and qPCR to screen healthy newborns. Seven confirmatory articles used a more comprehensive complementary biochemical testing such as metabolic profiling and blood biochemistry analyses in parallel with or as a reflex test for DNA-based tests to confirm a diagnosis in a newborn screened positive [24,25,27,28,29,32,45]. For metabolic profiling and blood biochemistry analysis, tests such as mass spectrometry (MS)–MS, liquid chromatography (LC)–MS/MS, gas chromatography–mass spectrometry, and high-performance liquid chromatography (HPLC) were among the tests used [24,27,29,44,45].

### 3.8. Genes and Conditions Included

The number of genes included ranged from 2 to 1514 and 35 to 306 in screening and confirmatory articles, respectively (Supplementary File S3). Four confirmatory articles did not provide data regarding the number of genes, but indicated that 11 [37], 16 [28], 17 [26], 18 [44] conditions were included in the panels used. qPCR used in screening (*n* = 2) and MassARRAY used for confirmatory testing (*n* = 35) included the fewest genes tested [8,30].

### 3.9. Gene List Comparison Across Screening Articles

**Figure 4:**
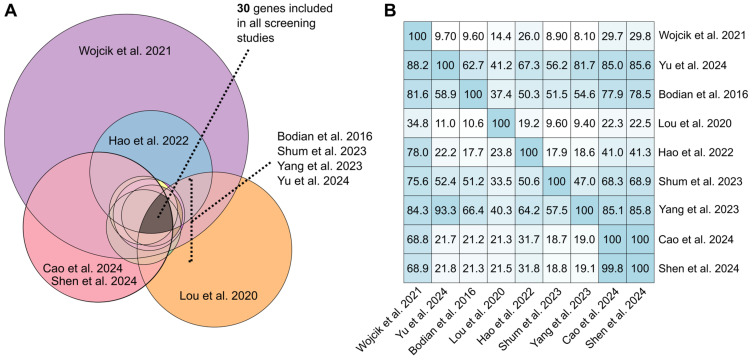
(**A**) Euler diagram of the overlap of genes tested in the nine reviewed screening articles. (**B**) Heatmap showing the percentages (%) of overlap in genes tested in the nine reviewed screening articles [21,23,33,34,35,36,38,39,40].

For screening, only 30 genes (*ACADM*, *ACADSB*, *ACADVL*, *ACAT1*, *ARG1*, *ASL*, *ATP7B*, *AUH*, *BCKDHA*, *BTD*, *CPS1*, *CPT1A*, *ETFA*, *FAH*, *GALC*, *GCDH*, *GLA*, *GLDC*, *HADHA*, *HBB*, *HLCS*, *HMGCL*, *IL2RG*, *MAT1A*, *MLYCD*, *NPC1*, *OTC*, *PCCA*, *SLC25A13*, and *SLC25A20*) (Figure 4A) were commonly tested across the nine articles with available lists of more than 2 genes [21,23,33,34,35,36,38,39,40] (Supplementary File S4). In sum, 23 (77%) of the 30 genes are associated with conditions under the Recommended Uniform Screening Panel (RUSP) list of core (*n* = 14) and secondary (*n* = 9) conditions. The *ATP7B*, *CPS1*, *GALC*, *GLA*, *GLDC*, *NPC1*, and *OTC* genes are associated with non-RUSP listed conditions such as Wilson’s disease, carbamoyl phosphate synthetase I deficiency, Krabbe’s disease, Fabry disease, glycine encephalopathy 1, Niemann–Pick disease, and ornithine transcarbamylase deficiency, respectively, were also commonly tested (Supplementary File S4).

Four screening articles mentioned inclusion of late-onset conditions [22,23,35,38] and one article reported inclusion of pediatric pharmacogenetic genes [23].

### 3.10. Detection of VUS, Low/Incomplete-Penetrance Variants, Carrier State of Technologies

Variants of uncertain significance (VUS), variants with low/incomplete penetrance, and variants in heterozygous/carrier states were detected by WGS, WES, and TGS (Table 2). For qPCR and MassARRAY, VUS and low/incomplete-penetrance variants were not detected, but heterozygous carriers were.

### 3.11. Cost

Two screening articles provided information about costs and cost per positive newborn screen, both for TGS. In a Chinese study by Yu et al., the cost of a NeoSeq report [36] based on a TGS panel was RMB 1600 (USD 219). The cost was higher compared with biochemical screening (RMB 180 or USD 25) and between the cost of an NGS panel (RMB 2500 or USD 343) and Sanger sequencing (RMB 500 or USD 69) [36]. Shum and colleagues reported that the cost per positive screen using TGS was USD 7418, which was considered more cost-effective compared with a cost of USD 92,400 per positive screen when multiplex PCR was introduced for SMA and SCID [34].

## 4. Discussion

This scoping review covered 26 published articles and identified five different multiplexable, high-throughput, DNA-based technologies used in screening (*n* = 12) and confirmatory (*n* = 14) testing of newborns—WGS, WES, TGS, qPCR and MassARRAY—of which TGS is the most commonly used technology (*n* = 19, 73.08%). Generating a time trend showed an increase in the number of published screening articles from 2022 to 2024 in contrast to confirmatory articles, reflecting the increasing role of DNA-based tests, specifically NGS, in NBS [14]. Evidence extracted was mainly on technical aspects such as sensitivity, specificity, and gene and disease coverage. Data regarding TAT, suitability of DBS, and variant detection and interpretation limitations were also recorded.

In the reviewed articles, sensitivity and specificity values were provided for screening using TGS (sensitivity: >94.17% and >99%, specificity: 100%) [34,36]. It was shown that if TGS was combined with biochemical analysis, the sensitivity (99.17%) and specificity (100%) improved [36]. Although this was based on the information provided by one article only, this trend has been seen in other studies showing that parallel genetic and traditional NBS improve accuracy of testing newborn disorders [46,47,48]. A combined approach has proven to be beneficial for conditions with variable penetrance, such as G6PD deficiency, and disorders not accurately detected by biochemical NBS, e.g., type I citrullinemia and cblC-methylmalonic acidemia. Here, a combined approach allows for rapid diagnosis due to the availability of data for genotype–phenotype correlations [46].

For gene and disease coverage, genes included in NGS technologies (*n* = 38 to 1514) are higher than those of qPCR (*n* = 2) [8] and MassARRAY (*n* = 35) [30], as expected. However, the important role of simpler and cheaper methods such as qPCR and MassARRAY cannot be discounted. This is particularly relevant for countries that are not keen to expand NBS via NGS due to limited resources and national restrictions stemming from ethical and sociological issues that NGS may raise. In these cases, these two methods are still practical options to consider. To achieve the primary goal of newborn screening, which is early detection and treatment, a short window of opportunity between birth and onset of symptoms must be considered in checking for the TAT of tests that will be used for screening newborn conditions. TATs were provided by the seven screening articles included in the review; however, none of those met the recommended same-day [49] or 7-day (from birth including collection and transport) TAT [50] for some conditions that need urgent results, except for SCID, where 28 days is allowed as its clinical presentations appear at 6 months of age, allowing a wider window for screening and confirmation [8,51]. Interestingly, two articles in this review used WES with a significant difference in TATs: one study that tested neonates screened for inborn errors of metabolism in a confirmatory test setting obtained results in 7 days [31], while the other study tested a population that included 80% healthy newborns in a screening setting had a TAT of 112 days [23]. Distinct symptoms or clinical conditions are known to assist in variant interpretation [52], hence for WES used for confirmatory testing, where the child with symptoms is presented to the clinician, it most probably contributed to a shorter TAT. Technically, one of the shortest TAT for WGS reported until now was an average of 39.80 h in a clinical setting where patients tested were critically ill pediatric patients with a well-described phenotype [53]. These kinds of pipelines may be explored and tailored for NBS to speed up the follow-up process.

The use of DBS in screening of newborns is viewed as crucial due to its ease of collection, transport, and storage, which is critical in general, but even more so in under-resourced settings [54]. In addition to its use in traditional NBS, DBS has expanded its use to DNA-based testing, specifically as a source of gDNA for newborn genetic testing and sequencing. In the articles reviewed, DBS was effectively used as a source of gDNA for WES, TGS, and qPCR, but no article was included that used DBS for WGS and MassARRAY, although in theory this should be feasible. The feasibility of using DBS as a DNA source for WGS has been proven in several studies already [55,56,57,58,59], with one study using 20-year-old archived DBS [55]. However, one of the first studies that successfully showed that DBS can be used for WGS was published in 2021 [59], which possibly explains why the WGS article included in this review did not use DBS, as it was published in 2016 [21]. For MassARRAY, to the authors’ knowledge, no studies have been published using DBS as a source of gDNA. The intended use of MassARRAY in the article included was for confirmatory testing using peripheral whole-blood samples. To allow the use of MassARRAY for screening newborn conditions, specifically in underprivileged and logistically challenged settings, optimization of the use of DBS for MassARRAY is suggested.

Mainly, accuracy in variant detection and interpretation are among the technical concerns raised with NGS use in screening newborn conditions [1,17], in contrast to qPCR and MassARRAY. The main differences between different NGS technologies were already published elsewhere [15,60], where it was shown that WGS is superior in sequencing coverage when compared with WES and TGS, though it produces substantially larger amounts of data to interpret and store. In this review, for each identified multiplexable DNA-based technology, the reported technique-specific limitations or shortcomings across multiple techniques were listed, including the complementary tests that were used to address the limitations. Regardless of the identified technology, the encountered limitations dictate if a complementary DNA-based and non-DNA-based test needs to be used. Examples mentioned are Sanger sequencing for poorly covered regions [27], MLPA to detect missed CNVs [33,41,44], and tandem mass spectrometry to differentiate between HbS/A and sickle cell disease [8]. These examples suggest that the most optimal DNA technique for a given condition depends on the specific disorder and the associated characteristics of the genes involved. Hence, there may be no single technique that performs optimally for all conditions in the context of screening, and therefore complementary testing will continue to play an important role.

Furthermore, the main challenge in traditional biochemical NBS is that screening and confirmatory testing of conditions is primarily based on alterations in metabolic profile in the absence of clinical manifestations of the disorder at the time of testing [61]. Hence, it is beneficial that for both screening and confirmatory testing, complete data (biochemical and genetic) for screening and diagnosing a condition are both available. Due to the possible absence of (information on) clinical manifestations and due to intrinsic limitations of both types of tests (biochemical vs. genetic) and technologies (WGS vs. WES vs. TGS) used, availability of both biochemical and genetic results is beneficial. This is even more important for confirmatory testing where a DNA-based technology is used to confirm cases where a metabolic profile or genetic result is not clearly indicative of the condition. In these cases, it is crucial that the confirmatory test covers all possible causes of the disease involved, as this is needed to confirm or exclude the diagnosis and avoid misdiagnosis.

Although limited, ethical aspects pertaining to detection of non-actionable findings, late-onset diseases, and privacy of personal genomic data were also extracted from reviewed articles. Among the technologies identified, NGS technologies are particularly prominent, as a key advantage of these methods is their ability to collect an increased amount of genomic information. However, at the same time, these broader technologies also raise increased ethical concerns [1]. With the potential expansion of conditions tested in NBS programs through NGS use, issues about inclusion of conditions without treatment or management in panels, reporting of high-penetrance adult-onset conditions and pharmacogenetic-related variants, identification and reporting of VUS, carrier state, and low- or variable-penetrance variants, long-term storage and potential misuse of data, i.e., to discriminate in employment or insurance and breach of privacy, were among the ethical concerns reported [1,17,54].

In the nine reviewed screening articles with an available list of genes, 23% (*n* = 7) of the common genes included in the panels of projects were non-RUSP condition-associated genes. Particularly Wilson’s disease (caused by pathogenic variants in *ATP7B*), Fabry disease (*GLA*), and glycine encephalopathy 1 (*GLDC*) were reported to have disease onset variability and late-onset cases [14]. Krabbe’s disease caused by *GALC* variants, and glycine encephalopathy 1 are currently managed, but considered untreatable [62,63]. One of the reviewed studies reported inclusion of pediatric pharmacogenetic genes, which represent a distinct category from genes associated with conditions generally included in newborn screening programs [23]. These discrepancies in genes and conditions included in current newborn sequencing projects were pointed out already in another publication [14]. It is important to emphasize that inclusion of late-onset, non-treatable disease, pharmacogenetic, and predisposition genes in newborn screening is currently controversial. However, inclusion of conditions in programs in a particular country or state depends on the country’s specific needs, such as disease prevalence and access to treatment or medication, as well as restrictions such as healthcare resources. Selecting genes and conditions to prioritize in newborn DNA sequencing projects may be based on the predicted list inclusion [14], the suggested panel of RUSP conditions [1], the European Society of Human Genetics (ESHG) recommendation for selection of conditions [1,64] and the Wilson and Jungner criteria.

Detection of VUS, carrier status, and low- or variable-penetrance variants by all NGS technologies (Table 2) was also reported in the reviewed articles. The detection of a heterozygous carrier state is possible for all identified technologies, and the identification of VUS and carrier state is inevitable with the use of NGS technologies [1]. Although this can be limited by applying stringent in vitro variant filtering to report only variants of interest, the availability of this information and reporting of these types of variants to parents have been among the ethical concerns posed with the use of NGS. The uncertainty of the clinical significance of VUS and of low- or variable-penetrance variants creates indecision and limits the application of evidence-based treatment and monitoring [54]. Identification of carrier status similarly presents ethical dilemmas, as this information has no immediate benefit to the child and may be disclosed without the child’s consent. It is important to note, however, that for newborns with clinical symptoms for an autosomal recessive condition such as CF, identification of a heterozygous pathogenic variant is beneficial, as its detection would prompt further analysis and testing [65]. In some cases, such as in Fabry disease, some carriers also develop full clinical presentation [66]. Reclassification for VUS upon availability of data is also possible [67]. Drafting guidelines regarding identification, monitoring for reclassification, and reporting of these variants in the context of population screening should also be considered. It is important to consider in the guideline that all the knowledge about pathogenic variants (e.g., in ClinVar) comes from patient cohorts, and established pathogenic variants in cohorts may have variable expressivity/reduced penetrance when used in testing healthy populations, such as in an NBS setting.

Preselection of variants, genes, and conditions for inclusion in the panel during the design phase is possible for qPCR and MassARRAY where there are no large genomic sequences generated, whereas for the sequencing data generated by WGS (and to some extent by WES), although selection by variant filtering and prioritization of selected genes may be performed during bioinformatic analysis and interpretation, the sequencing data still hold large amounts of personal information that can be potentially misused in the future [1]. A guideline for data storage, accessibility and future access, and reanalysis and reuse of genomic data is suggested if NGS is to be considered for application in NBS.

Economic aspects such as cost and cost per positive screen if available were also mined from the 26 articles. In this review, only two articles (8%) provided information for *partial* economic evaluation, which reported cost [36] and cost per positive screen [34], both for TGS. The economic criteria for screening programs are not based solely on the cost of a genetic test per case and mutation detected, but also on expenses related to the analysis, the interpretation, and the follow-up procedures and overall impact on health outcome and downstream costs [68]. For decision-makers, *full* assessment of genetic services where health effects is also added to the cost, such as a cost-effectiveness analysis and a cost–utility analysis, are crucial to determine whether the benefits of new tests justify their expenditures [68,69]. Hence, while a decrease in the cost of NGS technology or other technologies or tests is relevant, alone it offers insufficient information for a thorough economic assessment of the potential advantages for NGS in a screening context. Cost-effectiveness studies conducted for WES [70,71] in neonates with suspected monogenic conditions and WGS [72] in critically ill newborns showed cost-saving and/or at least cost-neutral assessment. These were carried out in cohorts of suspected patients, while cost-effective studies on a larger-scale newborn screening setup are currently lacking.

### Strengths and Limitations

This scoping review has allowed an unbiased selection of published journal articles for review to identify and evaluate the different multiplexable, high-throughput DNA-based technologies and the suitability of their use in an NBS setting. However, due to strict selection criteria, a low number of articles per technology were included, particularly for WGS, qPCR, and MassARRAY, resulting in less available information for these technologies. Validation studies of these technologies were also excluded to focus more on the assessment of suitability of these technologies in screening and confirmatory settings of NBS. Several ongoing NBSeq projects published their plans or status updates, but were not yet completed at the of date of search and not included in our study [14]. Upon brief scanning of published articles from April 2024 to August 2025, four articles were found: two were not eligible for review based on the set criteria [73,74] and two that could have been included, but would not have significantly changed the main results of the review [75,76].

## 5. Conclusions

Evaluations of the technical aspects of technologies identified in this scoping review suggest that in terms of expanding disease and gene coverage, which offers new opportunities for newborn screening, WGS appears to have the greatest potential from a technical perspective, followed by WES and TGS. However, each of these technologies has distinct and shared limitations in variant detection and interpretation. Disease prediction based on DNA-based tests alone in asymptomatic newborns will therefore remain complicated, and it will take time before these technologies can take on a leading role in NBS. In the meantime, complementary testing using DNA and biochemical tests, either parallel or reflex tests, may prove to be the best solution for screening. For confirmatory testing, combined testing using genetic and biochemical tests either as reflex or parallel testing is crucial to avoid misdiagnosis. The role of simple methods such as qPCR and MassARRAY cannot be dismissed, as the application of these technologies is still a practical option for under-resourced settings funded by public health and in the complex screening organization that NBS currently is.

From an ethical and economic perspective, the large DNA sequence data generated via WGS and WES presents challenges in terms of long-term data storage and privacy. Further research involving a full economic assessment, i.e., cost-effectiveness, of NGS in the NBS setting is needed.

## Figures and Tables

**Table 1:** Limitations in variant detection and interpretation and proposed solutions of technologies identified from the 26 reviewed articles.

Technology	Types of Variants Detected	Limitation	Complementary DNA-Based Technology/Methods Used to Address the Limitation (If Available)
WGS [21]	SNVs, ^1^ small indels	**Intrinsic** Requires variant confirmation	Sanger sequencing, clinical exome
		**Data quality** Some genes were not fully covered, e.g., *GBA*, *HBA1*, *CFTR*, *MYCLD* and *FOXE1*	
		**Data analysis and interpretation** High numbers of results of uncertain significance	
		Ambiguity of mapping of highly homologous genes and pseudogenes, e.g., *CYP21A2* and *CYP21P*	Entrez mapping
WES [22,23,31,37]	SNVs, ^1^ small indels, indels, large genomic rearrangements	**Intrinsic** Requires variant confirmation	Sanger sequencing, Sanger–Coulson method, droplet PCR
		Missed detection of variants in deep intronic regions	Sanger sequencing
		Restricted detection of large CNVs	
		**Data quality** Detection of variants in genomic regions with low read depth in ES are not confirmed using orthogonal methods, e.g., poor coverage of exon 1 of *MCCC2*	
		Low exome coverage which resulted to missed detection of deletion	
		**Data analysis and interpretation:** Missed detection of variants due to the limitations of set reporting standards, e.g., hemoglobin variant FAV	
		Limitation in variant interpretation, e.g., false-negative interpretation of a supposed splice site variant	Transcriptional profile analysis
		Identifying pathogenic variants that are not clearly protein-altering remains challenging.	
		Missed detection of variants in genes not included in panels, as these genes were rarely associated or not known to be associated with the disorders	Sanger sequencing
TGS [24,25,26,27,28,29,30,32,33,34,35,36,38,39,40,41,42,43,44,45]	SNVs, ^1^ small indels, indels (CNVs)	**Intrinsic** Requires variant confirmation	Sanger sequencing
		Missed detection of large CNVs	MLPA, long-range PCR
		Phasing of variants identified could not be determined	Sanger sequencing of parents’ gDNA
		Predominant coverage of exonic regions only, intronic and regulatory regions not covered	
		Difficulties associated with detection of complex structural variants, e.g., large fragment deletions/duplications and complicated structural rearrangements	MLPA for deletion and duplications
		**Data quality** Poorly covered exons due to subpar performance of NGS libraries	Sanger sequencing, further protocol optimization
		**Data analysis and interpretation** Technical challenge in analyzing highly homologous genes and pseudogenes, e.g., the highly identical pseudogene of *CYP21A2* makes it difficult to assess the accuracy of sequencing	
		Technical challenge in analyzing highly homologous regions, e.g., *HBA*, *HBB*, *SMN1*, including CNV detection in those regions	Custom probe design, fluorescent PCR melting curve method, MLPA Special bioinformatics method used in data analysis
qPCR [8]	SNVs, deletion, quantity of T-cell receptor excision circles	**Intrinsic** Requires additional testing to differentiate carrier from pathogenic variants, i.e., HbS/A carrier state vs. sickle cell disease Limited variants/conditions were checked	^2^ Non-DNA-based method was used
^3^ MassARRAY [30]	SNVs, small indels	**Intrinsic** Limited genes included in the panel	TGS and Sanger sequencing

CNVs, copy number variants; DBS, dried blood spot; ES, exome sequencing; gDNA, genomic deoxyribonucleic acid; MLPA, multiplex ligation-dependent probe amplification; qPCR, quantitative polymerase chain reaction; SNVs, singe-nucleotide variants; TGS, targeted gene sequencing; WES, whole-exome sequencing; WGS, whole-genome sequencing. ^1^ Small indels are insertion or deletions involving 1–50 nucleotides only. ^2^ The non-DNA-based method used was tandem mass spectrometry. ^3^ This technology was used in combination with TGS in the article by Yang et al. [30].

**Table 2:** Detection of VUS, low/incomplete-penetrance variants, and carrier status by identified technologies.

	VUS	Low/Incomplete-Penetrance Variants	^1^ Heterozygous Carrier Status
WGS [21]	Yes	Yes	Yes
WES	Yes [23]	Yes [23]	Yes [23,31]
TGS	Yes [24,25,26,29,32,33,34,38,40,42,43,45]	Yes [29,36,38,42]	Yes [27,28,32,33,34,35,38,39,40,41,42,44]
qPCR [8]	No	No	Yes
MassARRAY [30]	No	NR	Yes

qPCR, quantitative polymerase chain reaction; NR, not reported; TGS, targeted gene sequencing; VUS, variants of uncertain significance; WES, whole-exome sequencing; WGS, whole-genome sequencing. ^1^ Detection of one pathogenic variant for autosomal recessive condition for both male and female newborn and X-linked autosomal recessive condition for female newborn.

## Data Availability

The original contributions presented in this study are included in the article/Supplementary Material. Further inquiries can be directed to the corresponding author.

## Supplementary Materials

The following supporting information can be downloaded at https://www.mdpi.com/article/10.3390/ijns11040104/s1. Supplementary File S1: Extracted raw data; Supplementary File S2: Complete search strategies; Supplementary File S3: Characteristics of the 12 selected screening and 14 selected confirmatory articles; Supplementary File S4: Gene list included per article.

## Abbreviations

The following abbreviations are used in this manuscript.

DBS	dried blood spot
NGS	next-generation sequencing
WES	whole-exome sequencing
TGS	targeted genome sequencing
qPCR	quantitative polymerase chain reaction
WGS	whole-genome sequencing
SNV	single-nucleotide variant
CNV	copy number variant
PCR	polymerase chain reaction
gDNA	genomic deoxyribonucleic acid
NBS	newborn screening
VUS	variant of uncertain significance
TAT	turnaround time
