# Supplementary material for: Multiplexable, High-Throughput DNA-Based Technologies in Screening and Confirmatory Testing of Newborn Conditions: A Scoping Review
Source: Int J Neonatal Screen. 2025 Nov 13;11(4):104. doi: 10.3390/ijns11040104 (PMC12641697; doi:10.3390/ijns11040104)
Supplement: Supplementary file 1 [file IJNS-11-00104-s001.zip › Supplementary S2 Complete Search Strategies.pdf]

Supplementary S2

Complete search strategies
OVID/Medline (April 18, 2024)

Ovid MEDLINE(R) ALL <1946 to April 17, 2024>

**1**	exp "Infant, newborn"/ or (newborn* or new-born* or newly-born* or neo-nat* or neonat* or infan* or baby or babies).ti,ab,kf.	**1,231,282**
**2**	exp "Infant, Newborn, Diseases"/ or (disorder* or condition* or disease* or illness* or congenital* or inborn*).ti,ab,kf.	**8,658,687**
**3**	exp "Neonatal Screening"/ or (screening* or testing* or primary-test* or preliminary-test* or initial-test* or 1st-line-test* or first-line-test* or confirmatory or diagnos* or tier-test* or secondary-test* or third-test* or follow-up-test* or followup-test* or 2nd-line-test* or second-line-test* or 3rd-line-test* or third-line-test*).ti,ab,kf.	**4,323,515**
**4**	exp "High-Throughput Screening Assays"/ or "High-Throughput Nucleotide Sequencing"/ or exp "Whole Genome Sequencing"/ or exp "Nanopore Sequencing"/ or exp "Multiplex Polymerase Chain Reaction"/ or (high-throughput-nucleotide-sequenc* or high-throughput-dna-sequenc* or whole-genome-sequenc* or wgs or wgss or whole-exome-sequenc* or wes or wess or next-generation-sequenc* or ngs or ngss or tngs*1 or illumina-sequenc* or ion-torrent-sequenc* or nanopore-sequenc* or multiplex-ligation-dependent-probe-amplificati* or mlpa*1 or optical-genome-map* or ogm*1 or high-throughput-screen* or high-throughput-biological-assay* or high-throughput-chemical-assay* or ion-proton-sequenc* or deep-sequenc* or massively-parallel-sequenc* or pyrosequenc* or high-throughput-sequenc* or complete-genome-sequenc* or multiplex-pcr or triplex-polymerase-chain-reaction or triplex-pcr).ti,ab,kf.	**252,545**
**5**	1 and 2 and 3 and 4	**3,666**
**6**	5 not (rna or rnas or ribonucleic-acid*).ti,ab,kf.	**3,550**
**7**	6 not (exp "Animals"/ not exp "Humans"/)	**3,510**

Embase.com (April 18, 2024)

**No.**	**Query**	**Results**
#9	#7 NOT #8	**4,445**
#8	#7 AND ('Conference Abstract'/it OR 'Conference Paper'/it OR 'Editorial'/it OR 'Letter'/it OR 'Note'/it)	**2,560**
#7	#6 NOT ([animals]/lim NOT [humans]/lim)	**7,005**
#6	#5 NOT ('rna'/exp OR rna:ti,ab,kw OR rnas:ti,ab,kw OR 'ribonucleic acid*':ti,ab,kw)	**7,104**
#5	#1 AND #2 AND #3 AND #4	**7,675**
#4	'high throughput screening'/exp OR 'high throughput sequencing'/exp OR 'whole genome sequencing'/exp OR 'multiplex polymerase chain reaction'/exp OR 'illumina sequencing'/exp OR 'semiconductor sequencing'/exp OR 'optical genome mapping'/exp OR 'massively parallel sequencing'/exp OR 'high throughput nucleotide sequenc*':ti,ab,kw OR 'high throughput dna sequenc*':ti,ab,kw OR 'whole genome sequenc*':ti,ab,kw OR wgs:ti,ab,kw OR wgss:ti,ab,kw OR 'whole exome sequenc*':ti,ab,kw OR wes:ti,ab,kw OR wess:ti,ab,kw OR 'next generation sequenc*':ti,ab,kw OR ngs:ti,ab,kw OR ngss:ti,ab,kw OR tngs*1:ti,ab,kw OR 'illumina sequenc*':ti,ab,kw OR 'ion torrent sequenc*':ti,ab,kw OR 'nanopore sequenc*':ti,ab,kw OR 'multiplex ligation dependent probe amplificati*':ti,ab,kw OR mlpa*1:ti,ab,kw OR 'optical genome	**431,817**

	map*':ti,ab,kw OR ogm*1:ti,ab,kw OR 'high throughput screen*':ti,ab,kw OR 'high throughput biological assay*':ti,ab,kw OR 'high throughput chemical assay*':ti,ab,kw OR 'ion proton sequenc*':ti,ab,kw OR 'deep sequenc*':ti,ab,kw OR 'massively parallel sequenc*':ti,ab,kw OR pyrosequenc*:ti,ab,kw OR 'high throughput sequenc*':ti,ab,kw OR 'complete genome sequenc*':ti,ab,kw OR 'multiplex pcr':ti,ab,kw OR 'triplex polymerase chain reaction':ti,ab,kw OR 'triplex pcr':ti,ab,kw	
#3	'newborn screening'/exp OR screening*:ti,ab,kw OR testing*:ti,ab,kw OR 'primary test*':ti,ab,kw OR 'preliminary test*':ti,ab,kw OR 'initial test*':ti,ab,kw OR '1st line test*':ti,ab,kw OR 'first line test*':ti,ab,kw OR confirmatory:ti,ab,kw OR diagnos*:ti,ab,kw OR 'tier test*':ti,ab,kw OR 'secondary test*':ti,ab,kw OR 'third test*':ti,ab,kw OR 'follow up test*':ti,ab,kw OR 'followup test*':ti,ab,kw OR '2nd line test*':ti,ab,kw OR 'second line test*':ti,ab,kw OR '3rd line test*':ti,ab,kw OR 'third line test*':ti,ab,kw	**6,211,730**
#2	'newborn disease'/exp OR disorder*:ti,ab,kw OR condition*:ti,ab,kw OR disease*:ti,ab,kw OR illness*:ti,ab,kw OR congenital*:ti,ab,kw OR inborn*:ti,ab,kw	**12,510,429**
#1	'newborn'/exp OR newborn*:ti,ab,kw OR 'new born*':ti,ab,kw OR 'newly born*':ti,ab,kw OR 'neo nat*':ti,ab,kw OR neonat*:ti,ab,kw OR infan*:ti,ab,kw OR baby:ti,ab,kw OR babies:ti,ab,kw	**1,420,747**

Clarivate Analytics/Web of Science Core Collection (April 19, 2024)

**6**	#5 NOT TS=("rna" OR "rnas" OR "ribonucleic-acid*")	**2,834**
**5**	#1 AND #2 AND #3 AND #4	**2,975**
**4**	TS=("high-throughput-nucleotide-sequenc*" OR "high-throughput-dna-sequenc*" OR "whole-genome-sequenc*" OR "wgs" OR "wgss" OR "whole-exome-sequenc*" OR "wes" OR "wess" OR "next-generation-sequenc*" OR "ngs" OR "ngss" OR "tngs*" OR "illumina-sequenc*" OR "ion-torrent-sequenc*" OR "nanopore-sequenc*" OR "multiplex-ligation-dependent-probe-amplificati*" OR "mlpa*" OR "optical-genome-map*" OR "ogm*" OR "high-throughput-screen*" OR "high-throughput-biological-assay*" OR "high-throughput-chemical-assay*" OR "ion-proton-sequenc*" OR "deep-sequenc*" OR "massively-parallel-sequenc*" OR "pyrosequenc*" OR "high-throughput-sequenc*" OR "complete-genome-sequenc*" OR "multiplex-pcr" OR "triplex-polymerase-chain-reaction" OR "triplex-pcr")	**266,497**
**3**	TS=("screening*" OR "testing*" OR "primary-test*" OR "preliminary-test*" OR "initial-test*" OR "1st-line-test*" OR "first-line-test*" OR "confirmatory" OR "diagnos*" OR "tier-test*" OR "secondary-test*" OR "third-test*" OR "follow-up-test*" OR "followup-test*" OR "2nd-line-test*" OR "second-line-test*" OR "3rd-line-test*" OR "third-line-test*")	**5,074,088**
**2**	TS=("disorder*" OR "condition*" OR "disease*" OR "illness*" OR "congenital*" OR "inborn*")	**12,474,415**
**1**	TS=("newborn*" OR "new-born*" OR "newly-born*" OR "neo-nat*" OR "neonat*" OR "infan*" OR "baby" OR "babies")	**982,182**
