# Supplementary material for: Multiplexable, High-Throughput DNA-Based Technologies in Screening and Confirmatory Testing of Newborn Conditions: A Scoping Review
Source: Int J Neonatal Screen. 2025 Nov 13;11(4):104. doi: 10.3390/ijns11040104 (PMC12641697; doi:10.3390/ijns11040104)
Supplement: Supplementary file 1 [file IJNS-11-00104-s001.zip › Supplementary S3 Characteristics of Articles.pdf]

Supplementary S3

Table S3.1. Characteristics of the 12 selected screening articles.

Author, Year	Setting	Technology used (*Sequencing approach for NGS technology)	Brief description (*Analysis approach for NGS technology)	No. of samples	Classification of newborns
Bodian et. al., 2016	Virginia, USA/1 hospital, 2011-2014	WGS	Genome sequencing and in silico gene filter for analysis	1,696	Ancestrally diverse cohort of healthy infants (and parents)
Roman et. al., 2020	North Carolina, USA/prenatal and pediatric clinics	WES	Exome sequencing and in silico gene filter for analysis	106	"Well child" newborns (61) and children diagnosed with metabolic disease (17) and hearing loss (28)
Wojcik et. al., 2021	Massachusetts, USA/3 hospitals	WES	Exome sequencing and in silico gene filter for analysis	159	Healthy (80% of the study population) and admitted newborns
Cao et. al., 2024	China/5 hospitals, October 2019 to September 2021	TGS	Custom targeted exome capturing followed by sequencing, in silico variant filtering	3,249	All newborns (including hospitalized infants and infants with abnormal results of conventional NBS)
Chan et. al., 2024	Hongkong/NBS laboratory of a hospital, September 1, 2021 to April 28, 2022	TGS	A custom-designed AmpliSeq panel for variants associated with the six included conditions	Screening, 1st tier: 22,883; 2nd tier: 421	NBS screened positive (primary biochemical markers exceeding cut-offs)
Hao et. al., 2022	China/8 hospitals, for pilot study - 1 hospital, October 2018 to June 2019	TGS	Targeted Next Generation Sequencing, in silico variant filtering	11,484 (3,923 for pilot study)	Newborns[1]
Luo et. al., 2020	Shanghai, China/NBS center of a hospital, September 2016	TGS	Targeted Next Generation Sequencing, in silico variant filtering	1,127	Randomly selected DBS from newborns[1]
Shen et. al., 2024	Huzhou, Zheijang Province. China/1 hospital, October 2022 to January 2023	TGS	Targeted Next Generation Sequencing, in silico variant filtering, detected variants	1,263	Newborns[1]

			validated by Sanger sequencing		
Shum et. al., 2023	Brisbane, Australia/Queensland NBS program, two week period, 2021	TGS	Targeted Next Generation Sequencing, in silico variant filtering	2,552	Consecutive DBS samples[1]
Yang et. al., 2023	China/12 hospitals, November 2020 to November 2021	TGS	Targeted Next Generation Sequencing, in silico variant filtering	21,442	Randomly collected samples from newborns[1]
Yu et. al., 2024	Changzhou, Jiangsu Province, China/1 hospital	TGS	Targeted Next Generation Sequencing, in silico variant filtering	1,012	Newborns (50 with confirmed genetic metabolic disorder, 222 with false positive results in biochemical screening, 740 healthy newborns)
Tesorero et. al., 2023	Heidelberg, Germany/NBS laboratory of a hospital, October 1, 2021 to March 31, 2022	qPCR	Quadruplex qPCR assay	26,779 and 69,236	Healthy Newborns

DBS, dried blood spot; qPCR, quantitative polymerase chain reaction; NBS, newborn screening; TGS, targeted gene sequencing; WES, whole exome sequencing; WGS, whole genome sequencing.

[1] These are newborns for 1st tier screening, hence, considered “healthy” at the time of testing.

Table S3.2. Characteristics of the 14 selected confirmatory articles.

Author, Year	Setting	Technology used *(*Sequencing approach for NGS technology)*	Brief description *(*Analysis approach for NGS technology)*	No. of samples	Classification of newborns
Luo et. al., 2024	Zhuzhou, Hunan Province, China/1 hospital, 2019-2022	WES	Exome sequencing with in silico variant filtering, confirmation via Sanger sequencing	Screening: 90,829; Confirmatory: 1,067	Suspected positive
Navarrete et. al., 2019	Spain/8 NBS centers, January 2012 to April 2017	WES	Exome sequencing and in silico gene filter for analysis	Screening: Not indicated; Confirmatory: 141	IEM screened newborns
He et. al., 2023	Nanning, Guangxi Province, China/NB IEM screening, July 1, 2019 to December 31, 2021	TGS	PCR amplification of target regions followed by sequencing, confirmation via Sanger sequencing	Screening: 16,207; Confirmatory: 33	Tandem Mass Spectrometry screened positive cases
Lampret et. al., 2020	Slovenia/Slovenia Expanded NBS program	TGS	Targeted Next Generation Sequencing, in silico variant filtering	Screening: 15,064; Confirmatory: 68 with positive results (and additional from 202 that turned out positive after 2nd testing)	NBS screened positive
Lin et. al., 2019	Quanzhou, Fujian Province, China/NBS center of a hospital, January 2014 to November 2018	TGS	Targeted Next Generation Sequencing with in silico variant filtering, confirmation via Sanger sequencing and/or MLPA	Screening: 364,545; Confirmatory: 4,809	Newborns with clear aberrant initial results
Mao et. al., 2020	Ningxia, China/neonatal center of a hospital, 2016 to 2019	TGS	Targeted Next Generation Sequencing, in silico variant filtering	Screening: 189,354; Confirmatory: 49	Confirmatory positive newborns and referred for genetic analysis
Men et. al., 2023	Jiangsu Province, China/neonatal disease screening subcenter, January 2015 to June 2021	TGS	Targeted Next Generation Sequencing, in silico variant filtering	Screening: 245,194; Confirmatory: 86	IEM screened (few of screened positive underwent differential diagnosis)

Smon et. al., 2018	Slovenia/Slovenia NBS program, 2013-2014	TGS	Targeted Next Generation Sequencing, in silico variant filtering	Screening: 10,048; Screening: 80	Screened with IEM for metabolic follow up.
Tan et. al., 2021	Liuzhou, China/Lizhou NBS center, December 2012 to June 2020October 1, 2021 to March 31, 2022	TGS	Targeted Next Generation Sequencing, in silico variant filtering	Screening: 111,986 healthy and 7,461 hospitalized high-risk infants; Confirmatory: 2,275	Suspected positive cases
Wang et. al., 2019	Suzhou, China, April 2014 to December 2018	TGS	Targeted Next Generation Sequencing, in silico variant filtering	Screening: 401,660; Confirmatory: 138	Newborns with one of the targeted IEM and referred for genetic analysis
Yang et. al., 2019	Jiangsu Province, China/3 hospitals, January 2014 to June 2018	TGS	Targeted Next Generation Sequencing, in silico variant filtering	Screening: 536,008; Confirmatory: 1,033	Positive after 2nd TMS, for differential and gene diagnosis
Zhang et. al., 2021	Xi'an, Shaanxi, China/neonatal screening center, January 2014 to December 2019	TGS	Targeted Next Generation Sequencing, in silico variant filtering	Screening: 146,152; Confirmatory: 305	Suspected positive referred to specialists for differential diagnosis and genetic testing
Zhang et. al., 2022	Suqian, China/expanded NBS, January 2016 to November 2020	TGS	Targeted Next Generation Sequencing, in silico variant filtering	Screening: 204,604; Confirmatory: 4,021	Suspected positive
[1]Yang et. al., 2020	Jinning, Shandong province, China, July 14, 2014 to December 31, 2018	MassARRAY	Selected variants were analysed using the MS-based MassARRAY assay	Screening: 514,234; Confirmatory: 233	Suspected newborn
		TGS	Analysed using dedicated software; Targeted Next Generation Sequencing, in silico variant filtering		

DBS, dried blood spot; IEM, inborn errorsies of metabolismm; NBS, newborn screening; NGS, next generation sequencing; TGS, targeted gene sequencing; WES, whole exome sequencing; WGS, whole genome sequencing.

[1] This article used two technologies namely, TGS and MassARRAY.
